# World TB Day — March 24, 2019

**DOI:** 10.15585/mmwr.mm6811a1

**Published:** 2019-03-22

World TB Day is observed each year on March 24. This observance provides an opportunity to raise awareness about tuberculosis (TB) and the measures needed to find, treat, and prevent this devastating disease.

In 2018, a provisional total of 9,029 TB cases were reported in the United States (incidence = 2.8 cases per 100,000 persons) (*1*), a decline from the 9,094 cases reported in 2017 and the lowest number of cases on record in the United States since reporting began in 1953. Increased diagnosis and treatment of latent TB infection remains essential to eliminating TB in the United States.

Worldwide, an estimated 10 million cases of TB were reported in 2017, a decline of 1.8% from 2016. Approximately 1.57 million persons died from TB in 2017, a 3.9% decrease from 2016 (*2*). The implementation of effective strategies, including expansion of TB preventive treatment, defined in the global setting as treatment for those who might be infected with TB and are at risk for progressing to TB disease, including persons living with human immunodeficiency virus infection, is necessary to reach global targets.

CDC is working with domestic and global partners to diagnose and treat TB in the United States and around the world. Additional information about World TB Day and CDC's TB activities is available at https://www.cdc.gov/tb/worldtbday.
